# Real World Human-LLM Interactions – Prospective blinded versus unblinded expert physician assessments of LLM responses to complex medical dilemmas

**DOI:** 10.1371/journal.pdig.0001278

**Published:** 2026-03-12

**Authors:** Itamar Ben Shitrit, Daphna Idan, Mark Volevich, Hadar Sharabi Goldenberg, Dolev Vaknin, Or Degany, Nitzan Abelson, Yair Binyamin, Raouf Nassar, Majd Nassar, Aviya Kedmi, Alexander Zlotnik, Sharon Einav

**Affiliations:** 1 Ben-Gurion Faculty of Health Sciences, Beer-Sheva, Israel; 2 Clinical Research Center, Soroka University Medical Center, Faculty of Health Sciences, Ben-Gurion University of the Negev, Beer-Sheva, Israel; 3 Technion - Israel Institute of Technology, Haifa, Israel; 4 Faculty of Medicine, Tel-Aviv University, Tel Aviv, Israel; 5 The Cheryl and Chaim Saban Children’s Hospital, Soroka University Medical Center, Beer-Sheva, Israel,; 6 Department of Anesthesia, Soroka University Medical Center and the Faculty of Health Sciences, Ben-Gurion University of the Negev, Beer-Sheva, Israel; 7 Pediatric Gastrointestinal Unit, Saban Children Hospital, Soroka University Medical Center, Beer-Sheva, Israel; 8 Maccabi Healthcare Services and Hebrew University Faculty of Medicine, Jerusalem, Israel; Peng Cheng Laboratory, CHINA

## Abstract

Current evaluations of large language models (LLMs) in healthcare have largely emphasized theoretical benchmarks and clinician oversight, with limited exploration of real-world physician-AI interaction. In this two-stage prospective study, we assessed physician satisfaction with LLM-generated responses to real clinical queries. This study did not evaluate clinical accuracy, patient outcomes, or patient safety. In the first unblinded stage, physicians used three models - a general-purpose model (GPT-4o), a reasoning-focused model (GPT-o1), and a healthcare-specific model (OpenEvidence) - to address 25 clinical dilemmas - and rated the quality of the responses. In the second blinded stage, the same physicians evaluated responses generated either by an LLM or by a human alone, without knowledge of the source. Across 100 real-world medical responses, median physician scores on a 5-point Likert scale were comparable between unblinded and blinded evaluations (p = 0.90). Satisfaction was not associated with physicians’ resistance to change, nor did it correlate with the accuracy or relevance of cited literature. These findings suggest that physicians did not favor information generated by LLMs over externally provided responses, and that clinician satisfaction alone may not serve as a reliable proxy for validating decision support tools.

## Author summary

Large language models (LLMs) are increasingly being explored as decision support tools in clinical medicine, yet little is known about how physicians experience them when used for real patient care. In this study, we asked physicians to use three LLMs to help address real clinical dilemmas encountered in their practice, and to rate the quality of the responses. In a second phase, physicians evaluated responses without knowing whether they were generated by an LLM or a human, and their satisfaction scores were similar in both conditions. Notably, satisfaction did not correlate with the accuracy of citations provided in the responses, and physicians’ general resistance to change did not influence their ratings. These findings suggest that physician satisfaction is an insufficient proxy for the actual quality of LLM-generated clinical content. As LLMs move closer to routine clinical use, relying on clinician satisfaction surveys as a validation method may be potentially misleading when assessing whether these tools are appropriate for supporting medical decision-making.

## Introduction

Large language models (LLMs) are increasingly being suggested as nearing the ripening point for adoption in healthcare settings as decision support tools. Tools such as OpenEvidence [1,2] and Perplexity [3] are being promoted within healthcare settings as healthcare-specific LLMs designed to synthesize evidence-based information to support clinical decision-making with literature-based clinical insights. While these technologies are increasingly deployed in clinical environments, regulatory, legal, and ethical frameworks emphasize the importance of maintaining a human in the loop, and clinicians are expected to retain oversight and accountability for AI-assisted decisions [4–6].

The United States Food and Drug Administration (FDA) guidance highlights the need for human evaluation in the integration of AI-based clinical decision support systems [7]. However, the FDA guidance also acknowledges the importance of evaluating the human-LLM team. Yet current literature on the evaluation of LLMs in healthcare primarily focuses on clinician oversight on theoretical LLM benchmarks but rarely examines the dynamics of human-LLM interaction [8]. This leaves a critical information gap in assessing how clinicians supervise LLM outputs [8]. That many of the publications assessing the quality of LLM performance use proxies of physician satisfaction [9–11] suggests that satisfaction is considered a good indicator of quality.

In this two-stage prospective study, we aimed to address this question by evaluating physician satisfaction with LLM responses to real-world clinical queries. First, physicians directly queried an LLM regarding clinical dilemmas encountered in practice, and evaluated the models’ responses (unblinded evaluation). In the second stage, the same physicians evaluated responses generated either by different LLM models or written manually by a human researcher, without knowing the source of the response (blinded evaluation). We also examined whether satisfaction with the response was associated with the existence, relevance, and accuracy of the medical literature cited in the response and with physician resistance to change.

Physician satisfaction is a subjective measure that is distinct from clinical validity. We chose to use citation quality as a proxy for clinical validity because of concerns about using LLM suggestions to guide treatment. We hypothesized that, physicians would be more satisfied with LLM responses generated while using the LLM themselves than with LLM responses generated by a third party. We further hypothesized that blinded evaluations would favor human-generated responses rather than LLM-generated responses. Finally, we hypothesized that satisfaction would be higher when citations were accurate and clinically relevant, and that higher resistance to change would be associated with lower satisfaction with LLM-generated responses.

## Methods

### Study design

This was a prospective, single-center study. During the initial, unblinded stage, physicians independently presented real‐world clinical dilemmas to the three LLMs, aware of which model produced each response, and then rated the responses.

**Fig 1:**
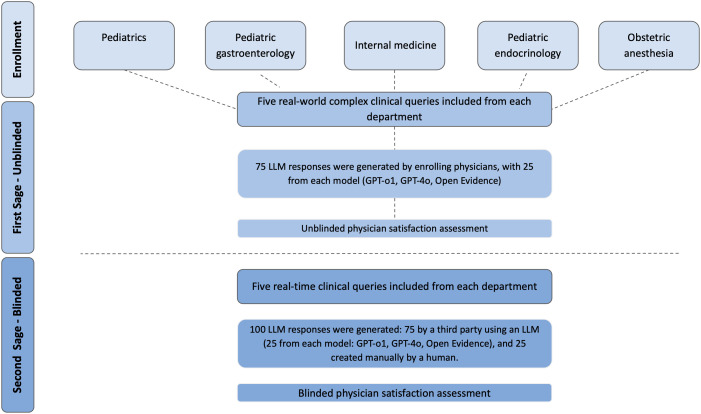
Study process flowchart. Illustration of the study stages – In the first stage, five departments each submitted five real-world clinical queries, yielding 75 responses generated with three LLMs (25 from each: GPT-o1, GPT-4o, Open Evidence), which were then assessed in an unblinded manner. In the second, blinded stage, the same five queries from each department produced 100 responses: 75 generated by a third-party user employing the three LLMs (25 from each model) and 25 created manually by a human, with all responses presented in a standardized format (layout, length and language style) and evaluated without knowledge of their source.

In the subsequent blinded stage, the same physicians evaluated four responses to a different set of clinical questions, without knowing their source: three responses generated by different LLM models (i.e., third party) and one manually prepared human researcher response (Fig 1). The human researchers were trained to perform patient-oriented literature reviews. For preparing the human responses, the researchers were allowed to consult all publicly available medical evidence sources (e.g., peer-reviewed literature indexed in PubMed, preprints, clinical trial registries, guidelines). However, only manual search for these sources was permitted; the use of LLM-based tools was explicitly prohibited when searching for content relevant to the preparation of human responses in the blinded phase. LLM responses were generated by a general-purpose model (GPT-4o), a reasoning-focused model (GPT-o1), and a healthcare-specific model (OpenEvidence). We report this study in accordance with STrengthening the Reporting of OBservational studies in Epidemiology (STROBE) recommendations [12].

### Clinical setting

The study was conducted at the Soroka University Medical Center (SUMC), a 1191-bed teaching hospital affiliated with Ben Gurion University. The SUMC is the sole provider of tertiary care in Israel’s southern region, which has a population of over 750,000 residents (8.2% of the Israeli population). As part of Clalit Health Services, the largest Israeli health insurance organization that insures over half the population, SUMC provides medical services to approximately 67% of the southern region’s residents, including a large Bedouin population [13].

### Use cases (population)

Only real-world dilemmas related to patient treatment were used. The dilemmas were presented as queries by practicing physicians from five domains (general pediatrics, pediatric endocrinology, pediatric gastroenterology, obstetric anesthesiology, and internal medicine). Two distinct sets of clinical dilemmas were used for the two study stages.

Five physicians evaluated the responses to five clinical dilemmas at each stage. In the first (unblinded) stage, physicians posed up to ten iterative questions per dilemma in each of the three models. The resulting outputs were not altered or edited by humans (total 75 responses). In the second (blinded) stage, physicians evaluated four responses for five different clinical dilemmas (total 100 responses).

Physicians were selected using purposive sampling, prioritizing mid-level physicians who treat complex cases daily but have no formal training with LLMs.

Included were queries regarding the clinical management of patients whose medical conditions presented a challenge for the physician. The queries were selected by each physician independently, based on their realization that to make a clinical decision, they must refer to the medical literature while taking into consideration unique individual case details.

Excluded were general queries on overarching topics unrelated to specific patient cases. The full list of queries included in the study is presented in Appendix A in S1 Text.

For the purpose of this study, the answers generated by either LLMs or humans to each query are termed “responses”. The LLMs used for generating responses were GPT-4o (by OpenAI), GPT-o1 (by OpenAI), and OpenEvidence 2.6.

### Outcome measures

The primary outcome was the overall quality and clinical usefulness of the response, as assessed by the physicians who submitted the queries (subjective). These metrics were rated on a 5-point Likert scale in both unblinded and blinded evaluations.

Secondary outcome measures included (1) Comparison of satisfaction in blinded only assessments (second stage) - human versus LLM responses. (2) Objective measures (i.e., the existence of the cited sources, their quality [journal impact factor and quartile], faithfulness [based on three criteria; correct publication year, accurate author list, and precise title matching]) and researcher assessment of the relevance of the citation to the case at hand (3) the association between source quality scores and clinician satisfaction; and (4) the association between physician resistance to change and satisfaction.

### Variables and their sources

Before study initiation, the participating physicians were requested to complete a Resistance to Change Scale Questionnaire (Appendix B in S1 Text) [14]. While submitting each query, the physicians also provided their impression of the complexity of the case at hand (simple, moderate, complex). Data were also collected on case characteristics.

The existence of each citation was verified through PubMed and Google Scholar searches or through a direct URL search for website sources. Journal impact factors and quartiles were obtained from Clarivate [15], or, if unavailable, from the official journal websites. Citation quality was assessed based on journal impact factor and quartile. Citation faithfulness was verified by correct publication year, accurate author list, and precise title matching. A citation was considered relevant when the content of its source matched the associated text and accordingly supplied information relevant to the clinical dilemma (Binary variable, Appendix C in S1 Text). To assess inter-rater reliability of reference relevancy, 10% of randomly selected references were independently reviewed by two blinded researchers. The resulting Cohen’s κ was 0.91, indicating almost perfect agreement per the Landis–Koch scale.

### Data collection

Before study initiation each physician underwent a brief, standardized training session on the use of the LLMs. In the unblinded phase the physicians were then allowed to run their queries on the LLMs either at work or at home. The LLMs were accessed using the standard web interfaces. The physicians also determined the stopping point for their investigation. After completing each investigation, they reported their satisfaction with the final LLM response using a 5-point Likert scale Satisfaction Questionnaire (Appendix D in S1 Text). This process was repeated with five different queries for each physician.

In the blinded stage, each physician submitted five new dilemmas to the research group and then received four responses at the same time: three generated by LLMs, and one written solely by a human. All responses were generated by trained researchers with LLM experience, and all responses were prepared in a similar format (layout, length and language style). The responses were then sent to the physician for evaluation. This process was also repeated with five different queries for each physician.

The quality of the responses was evaluated in parallel by the physicians (subjective measures) and by an independent researcher blinded to the source of the responses and to physician evaluations (objective measures). All data were collected using Microsoft Excel 2021.

### Bias

Bias may be introduced by the use of purposive sampling. We attempted to address preference bias through the selection of physicians with no prior experience with LLMs, as these still probably represent much of the mid-level physician population, and by providing standardized basic training in LLM use. To examine the possibility that dissatisfaction with the LLM response stems from hesitation to adopt a new technology, physician resistance to change was assessed. Outcome bias was addressed through physician blinding in the second stage of the study where physicians evaluated responses without knowledge of their source, allowing for comparison with unblinded evaluations where physicians were aware that LLMs were being used. As several of the authors are employees of the sponsor, analysis was performed by a blinded researcher uninvolved in data collection.

### Sample size

Overall 25 evaluations were paired with three comparators. To determine the minimal detectable difference allowed by our sample size, we performed calculations based on matched pairs (1:1 ratio). Based on the assumption that differences in report quality followed a normal distribution with a broad standard deviation of 1 (based on Likert scale assessments), we calculated that the study would be able to detect a true difference in the mean satisfaction from paired responses of ±0.522. With a narrower standard deviation of 0.5, the study would be able to detect a true difference in satisfaction from matched responses of ±0.261. Both calculations were conducted with a power of 0.8 and a Type I error probability of 0.05 for a two-sided test of the null hypothesis that there would be no difference in physician satisfaction. A 1:3 ratio was used to increase the study power.

### Handling of quantitative variables and statistical analyses

Missingness is reported. In the first stage, descriptive statistics were used to analyze case and participant characteristics, as well as the resistance to change questionnaire. Categorical data (e.g., sex, department) are reported as numbers and proportions (%). Clinician satisfaction was evaluated according to accepted practice [9,16]. Continuous variables (e.g., The main outcome measure of physician satisfaction, journal impact factors) are reported as mean ± SD and median (Interquartile Range [IQR]).

For the primary outcome measure, clinician satisfaction scores comparing unblinded versus blinded response conditions were analyzed using the Wilcoxon signed-rank test.

Secondary outcome analyses included: (1) comparison of clinician satisfaction scores within the blinded evaluation between human-generated and LLM-generated responses, with clinicians aware that one response was human-generated but blinded to which specific response using the Wilcoxon signed-rank test; (2) assessment of citation quality metrics generated solely by LLMs comparing unblinded and blinded evaluations, where categorical variables (e.g., citation relevance) were analyzed using Chi-square tests or Fisher’s exact test when appropriate, and continuous variables (e.g., journal impact factor) were analyzed using independent t-tests. Citation accuracy was reported as the percentage of non-hallucinated citations, citation faithfulness was presented as mean and standard deviation, and the proportion of faithful and relevant citations was calculated exclusively from verified, non-hallucinated citations; (3) evaluation of correlations between clinician satisfaction scores and objective quality metrics (e.g., impact factors, journal quartiles) using Pearson correlation coefficients; and (4) assessment of correlations between physicians’ median satisfaction scores and their resistance to change using Pearson correlation coefficients.

Subgroup analyses were performed to evaluate differences between human-generated and LLM-generated responses for each LLM compared to human responses in the blinded evaluation, as well as within-LLM comparisons of unblinded versus blinded evaluations for each specific LLM, across all secondary outcome measures.

All analyses were performed using R Studio (version 4.5.1), with statistical significance set at p < 0.05 (two-sided tests).

## Results

### Participants

**T**he participating physicians were aged 37 years (median, 33–41 years old). Their resistance to change scores ranged between 33–55 (maximal questionnaire range 17–102).

### Use cases

The dilemmas included patients aged 1 day to 70 years, and most were female. Approximately 57% of the dilemmas were classified as moderate in complexity, 25% as complex, and 18% as simple. The responses generated to the 50 dilemmas included: In the first (unblinded) stage - 75 responses (25 dilemmas) generated by the physicians using the three LLMs (GPT-4o, GPT-o1, and Open Evidence 2.6) and in the second (blinded) stage - 25 human responses and another 75 LLM responses were generated. Overall, 1,755 papers were cited in the responses, 411 in the human responses, 361 in GPT-4o responses, 441 in GPT-o1 responses, and 542 in OpenEvidence responses.

### Main outcome - Blinded versus unblinded physician satisfaction

We compared 75 unblinded evaluations with 75 blinded evaluations of responses generated by the three LLMs and found that, on a 5-point Likert scale (1 = lowest, 5 = highest), satisfaction did not differ significantly between conditions: median 4.0 (IQR 3.5–4.0) for the blinded evaluations and 4.0 (IQR 3.0–4.0) for the unblinded evaluations (p = 0.90; Table 1).

**Table 1:** Physician Satisfaction Scores: Physician-Generated LLM Responses (Unblinded) vs. Third-Party-Generated LLM Responses (Blinded).

Characteristic	Overall N = 150^1^	Blinded N = 75^1^	Unblinded N = 75^1^	p-value^2^
**Report is reliable for complex medical questions**	4.0 (4.0, 4.0)	4.0 (4.0, 4.0)	4.0 (3.0, 4.0)	0.4
**Information was relevant to medical inquiry**	4.0 (4.0, 5.0)	4.0 (4.0, 5.0)	4.0 (4.0, 5.0)	0.5
**Report included new information**	3.0 (2.0, 4.0)	3.0 (2.0, 4.0)	3.0 (2.0, 4.0)	0.6
**Information presented clearly**	4.0 (4.0, 4.0)	4.0 (4.0, 4.0)	4.0 (4.0, 5.0)	0.8
**Report was professionally written**	4.0 (4.0, 5.0)	4.0 (4.0, 5.0)	4.0 (4.0, 5.0)	0.14
**Report helped save time**	3.0 (2.0, 4.0)	4.0 (2.0, 4.0)	3.0 (2.0, 4.0)	0.6
**Report met expectations**	3.0 (3.0, 4.0)	3.0 (3.0, 4.0)	3.5 (3.0, 4.0)	>0.9
**Report length was appropriate**	4.0 (2.0, 4.0)	4.0 (2.0, 4.0)	4.0 (3.0, 4.0)	0.4
**Overall Median Score (All Questions)**	4.0 (3.5, 4.0)	4.0 (3.5, 4.0)	4.0 (3.0, 4.0)	0.9

1 Median (Q1, Q3)

2 Wilcoxon rank sum test

Subgroup analysis for within model satisfaction scores, unblinded vs. blinded, are presented in Table A in S1 Text.

#### Secondary outcome 1 - Comparison of blinded evaluations of LLM responses versus human responses.

In the blinded comparison, human responses received statistically significant higher median satisfaction scores than LLM responses for content reliability (p = 0.042), inclusion of new information (p = 0.034), and clarity of information presentation (p = 0.010), though overall median scores were similar (4.0 [3.5, 5.0] vs 4.0 [3.5, 4.0], p = 0.10) (Table 2).

**Table 2:** Physician Satisfaction Scores: Human-Generated Responses vs. Third-Party-Generated LLM Responses (Blinded).

Characteristic	Overall N = 100^1^	Human N = 25^1^	LLM N = 75^1^	p-value^2^
**Report is reliable for complex medical questions**	4.0 (4.0, 4.0)	4.0 (4.0, 5.0)	4.0 (4.0, 4.0)	0.042
**Information was relevant to medical inquiry**	4.0 (4.0, 5.0)	5.0 (4.0, 5.0)	4.0 (4.0, 5.0)	0.081
**Report included new information**	3.0 (2.0, 4.0)	4.0 (3.0, 4.0)	3.0 (2.0, 4.0)	0.034
**Information presented clearly**	4.0 (4.0, 4.0)	4.0 (4.0, 5.0)	4.0 (4.0, 4.0)	0.010
**Report was professionally written**	4.0 (4.0, 5.0)	5.0 (4.0, 5.0)	4.0 (4.0, 5.0)	0.063
**Report helped save time**	4.0 (2.5, 4.0)	4.0 (3.0, 4.0)	4.0 (2.0, 4.0)	0.3
**Report met expectations**	4.0 (3.0, 4.0)	4.0 (3.0, 5.0)	3.0 (3.0, 4.0)	0.050
**Report length was appropriate**	4.0 (2.0, 4.0)	4.0 (2.0, 4.0)	4.0 (2.0, 4.0)	0.5
**Overall Median Score (All Questions)**	4.0 (3.5, 4.0)	4.0 (3.5, 5.0)	4.0 (3.5, 4.0)	0.10

1 **Median (Q1, Q3)**

2 **Wilcoxon rank sum test**

**Fig 2:**
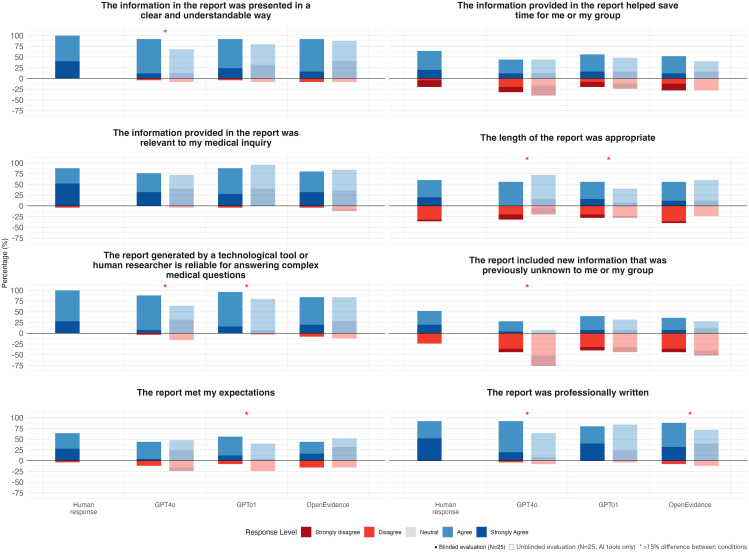
Blinded and unblinded evaluation of physician subjective assessment of large language model (LLM) and Human Responses. Data from unblinded and blinded subjective evaluation (N = 25 per group). Stacked bar charts show percentage distribution of Likert scale responses (1 = Strongly Disagree to 5 = Strongly Agree) for eight subjective quality measures. Side-by-side bars compare unblinded and blinded evaluations. Within blinded vs. unblinded model comparison a red dot indicates median ≥15% difference or more. Non-transparent bars show blinded evaluation where physicians evaluated responses without knowledge of whether they were human-created manual responses or third-party-generated LLM responses (GPT-4o, GPT-o1, or OpenEvidence), and transparent bars show unblinded evaluation of physician-generated LLM responses. Each group represents 25 evaluations of responses addressing the same medical questions.

Evaluations of each LLM versus the human-generated responses are detailed in Fig 2 and in Tables C (GPT4o), E (GPTo1) and G (OpenEvidence) in S1 Text.

#### Secondary outcome 2 - Blinded (n = 1091) versus unblinded LLM citation (n = 253) quality metrics.

No significant differences were observed in hallucination (citation existence) or faithfulness rates between unblinded versus blinded evaluation of LLM responses. However, there were more citations with high impact factors (median 4.6 [2.4, 10.1] vs 3.6 [2.3, 5.6], p < 0.001) and fewer first-quartile citations (63% vs 78%, p < 0.001) in unblinded versus blinded evaluation LLM responses. Unblinded evaluation of LLM responses included more guidelines, observational studies, RCTs, and book chapters, while blinded evaluation LLM responses included more systematic reviews. Finally, citations in the unblinded evaluation LLM responses were considered more relevant than those in the blinded evaluations (99% vs 86%, p < 0.001) (Table 3). This last difference stemmed mainly from GPTo1’s low relevance rate (66% vs 96%, p < 0.001) in blinded evaluations.

**Table 3:** Citation Quality Metrics: Physician-Generated LLM Responses (Unblinded) vs. Third-Party-Generated LLM Responses (Blinded).

Characteristic	Overall N = 1344^1^	Blinded N = 1091^1^	Unblinded N = 253^1^	p-value^2^
**Reference exists**	1,046 (78%)	839 (77%)	207 (82%)	0.12
**Year accurate**	852 (95%)	686 (94%)	166 (96%)	0.6
**Author accurate**	865 (97%)	696 (97%)	169 (98%)	0.6
**Journal accurate**	856 (96%)	680 (95%)	176 (98%)	0.069
**Overall accuracy score**	100.0 (100.0, 100.0)	100.0 (100.0, 100.0)	100.0 (100.0, 100.0)	>0.9
**Relevant**	910 (88%)	706 (86%)	204 (99%)	<0.001
**Publication type**				<0.001
Book/Chapter	21 (1.6%)	11 (1.0%)	10 (4.0%)	
Case Reports/Series	99 (7.4%)	87 (8.0%)	12 (4.7%)	
Guidelines/Recommendations	151 (11%)	105 (9.6%)	46 (18%)	
Narrative Review	227 (17%)	184 (17%)	43 (17%)	
Observational Study	232 (17%)	182 (17%)	50 (20%)	
Other	364 (27%)	315 (29%)	49 (19%)	
Randomized Controlled Trial	35 (2.6%)	24 (2.2%)	11 (4.3%)	
Systematic Review/Meta-analysis	73 (5.4%)	69 (6.3%)	4 (1.6%)	
Website	136 (10%)	109 (10.0%)	27 (11%)	
**Journal quartile**				<0.001
1	622 (75%)	522 (78%)	100 (63%)	
2	139 (17%)	107 (16%)	32 (20%)	
3	48 (5.8%)	29 (4.3%)	19 (12%)	
4	18 (2.2%)	10 (1.5%)	8 (5.0%)	
**Impact factor**	3.8 (2.3, 6.0)	3.6 (2.3, 5.6)	4.6 (2.4, 10.1)	<0.001

1 n (%); Median (Q1, Q3)

2 Pearson’s Chi-squared test; Wilcoxon rank sum test; Independent t-test

The objective quality metrics for individual LLM responses as well as for human responses are provided in Tables B (GPT4o), D(GPTo1), F (OpenEvidence) in S1 Text.

#### Secondary outcome 3 - Physician satisfaction vs. report citation quality metrics.

**Fig 3:**
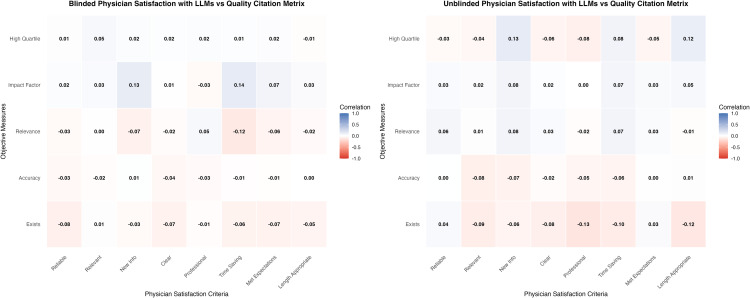
Heat maps of Pearson correlations between physician subjective satisfaction assessments and citation quality metrics, un blinded and blinded evaluations. Heat-map of Pearson correlation coefficients (r) linking eight physician satisfaction criteria (columns) with five objective quality metrics (rows) for AI models under blinded and unblinded conditions. The blinded condition includes evaluations where physicians were unaware of the AI source, while the unblinded condition includes evaluations where physicians created the responses themselves using an LLM. Human manually created data was excluded from both analyses. Cell hues range from red (negative correlation) through white (no correlation) to blue (positive correlation); exact r values are displayed within each cell. Correlations were computed using pairwise complete observations across all AI models within each blinding condition.

Only LLM responses were included in this analysis. Correlations between physician satisfaction and objective citations quality metrics (e.g., source relevance, impact factor) were weak and inconsistent. This finding was consistent in both unblinded and blinded evaluation LLM responses (Fig 3).

Physician satisfaction versus objective citations quality metrics for individual LLMs are provided in Fig A and Fig B in S1 Text.

Physician satisfaction across all LLMs is provided in Table H in S1 Text.

#### Secondary outcome 4 - Correlation between physicians’ median satisfaction score and their resistance to change.

No correlation was found between physician satisfaction versus physician resistance to change, in either unblinded or blinded evaluations (p = 0.25, p = 0.11, respectively).

## Discussion

In this study, physicians evaluated responses from LLMs (GPT-4o, GPT-o1, and OpenEvidence 2.6) when unblinded and blinded. LLMs were used to test physician satisfaction, with responses they generated through their own interactions with LLMs (unblinded), versus when receiving responses provided by a third party (blinded). No significant differences in overall physician satisfaction were found regardless of the manner of LLM use. However, physicians blinded to the source of the response evaluated human responses as having more reliable content, new information, and clarity of presentation than LLM responses. Human responses included more citations with lower impact factors that were more relevant to the case. When only LLM responses were studied, those generated by physician-LLM interactions had more citations with a high impact factor and more that were relevant to the case, than those provided by a third party. Physician satisfaction with responses correlated only weakly with objective citation quality metrics, regardless of the mode of response generation. Furthermore, physician satisfaction with responses did not seem associated with resistance to change.

The integration of artificial intelligence, including LLMs, into clinical workflows has followed two main approaches. A human-in-the-loop approach that theoretically ensures that clinicians remain in charge and potentially reduces LLM inaccuracies and hallucinations. This approach requires that the clinician review or validate model outputs before and during deployment. The second, fully embedded, approach positions the LLM as a routine partner in day-to-day clinical interactions, with minimal real-time oversight. Most studies focused on the former approach, often overlooking the complex dynamics that arise when LLMs are actively deployed in clinical practice [8].

The existing literature has largely focused on the evolution of specific LLMs. Consequently, it becomes increasingly important to move the focus beyond model-specific performance toward the broader and more enduring issue of human-LLM interaction. Unlike model-specific limitations, which may be quickly addressed by technical advances, the ways in which humans use, interpret, and adapt to LLM outputs represent ongoing challenges that will persist regardless of how fast the models themselves improve. Thus, findings tied to a single LLM version have only short-lived relevance.

Operationally, an LLM agent dedicated to validation of citations after report completion should be developed (e.g., essay evaluator, code reviewer, and/or safety assessor). To enhance safety and accountability in routine practice, LLM use as a clinical decision support tool should be embedded within a governance framework rather than relying solely on individual clinician judgment. Under this framework, the health system should provide the safest possible tools, including healthcare-specific LLMs that cite only verified, retrievable sources (e.g., PMID/DOI with accessible links) and flag uncertainty, missing inputs, and high-risk recommendations. The clinician should verify each recommendation in the context of the individual patient. A documentation step could confirm that the clinician reviewed the output critically and takes clinical responsibility for acting on it. Studies should be conducted on real-world LLM clinician use (which is already happening) and their correlation with the implementation of LLM suggestions and patient outcomes. Our finding that citation quality did not significantly affect physician satisfaction raises important concerns regarding potential automation bias. Citation metrics are probably more objective measures of quality than physician satisfaction, but neither equates clinical validity.

An expert panel discussing AI-human interaction concerns concluded that AI tools require trained teams capable of querying, interpreting, and challenging outputs. The panel recommended mapping human-AI interfaces [17]Click or tap here to enter text.. In the current study, physicians did not prefer responses they generated themselves using LLMs over information received from a third party, also generated by an LLM. These findings suggest that physicians are happy to use any tool that requires less manual search of the literature. A study showed that after 3 months of intermittent AI exposure, physicians had significantly lower adenoma detection rates when performing standard non-AI-assisted colonoscopy [18]. One might ask, what happens to the ability of the clinician to find quality relevant literature when using LLMs for three months.

Moreover, because LLMs ingest massive amounts of data from the open Internet during training, they are potentially exposed to misinformation [19]. This, combined with potential erosion of clinician abilities, raises further concerns regarding the placement of physicians and researchers as effective gatekeepers for validating information from decision support tools. Long-term reliance on these systems should not lean on physicians’ critical thinking abilities. In the current study, the quality of the sources cited did not correlate with physician satisfaction as well. For example, although 65% of the o1 model citations were hallucinated (did not exist), it did not affect physician satisfaction, suggesting automation bias [20,21] or the need to provide a response despite limited information [22]. Similarly, while OpenEvidence demonstrated superior source verification with no hallucinated references and outperformed general-purpose models, this improved performance did not translate to higher physician satisfaction. Even human-generated responses, which demonstrated both complete source verification and higher relevance rates, failed to achieve significantly higher satisfaction scores, underscoring the pervasive nature of automation bias and the limitations of subjective assessment in evaluating AI-generated medical content.

## Strengths and Limitations

The strengths of this study include its prospective design, blinding, use of multiple LLMs, and use of both subjective and objective performance metrics. A broad range of medical dilemmas from various specialties was assessed to better reflect the complexity and diversity of real-world clinical environments. The limitations of this study include its sample size, its single-center design, which may preclude generalizability and inclusion of a limited number of medical specialties. An additional limitation is the use of different clinical dilemmas in the unblinded and blinded stages, a limitation inherent to the study design (i.e., assessment by the same clinician). As per IRB demand, the responses were not relied upon for directing care, therefore data on clinical accuracy and safety were not collected; accordingly, the human comparator should be interpreted as a pragmatic reference rather than an outcome-based gold standard. We did not assess clinical accuracy, patient outcomes, and patient safety, which limits the applicability of our findings to real-world clinical practice. Finally, differences probably exist between physicians and non-clinical researchers in terms of training and information-seeking strategies. Such differences may have influenced both the interpretation of the research questions and the approach towards problem-solving, potentially affecting comparative performance.

## Conclusion

In this study physicians did not prefer information they generated themselves using LLMs over externally provided information. Physician satisfaction also did not seem associated with resistance to change. However, physician satisfaction did not correlate with objective citation quality, which suggests that physicians should not be leaned upon to serve as effective gatekeepers for validation of information from clinical decision support tools. As LLMs become more integrated into clinical practice, their deployment should be accompanied by rigorous validation frameworks and continuous performance monitoring to ensure their reliability.

## Supporting information

S1 Text Fig A. Heat maps of Pearson correlation coefficients between physician satisfaction and citation quality metrics in blinded evaluations Fig B. Heat maps of Pearson correlation coefficients between physician satisfaction and citation quality metrics in unblinded evaluations. Table A. Comparison of blinded and unblinded physician satisfaction for each model. Table B. Comparison of quality of citations (objective parameters) in GPT-4o and human responses (blinded evaluations). Table C. Comparison of physicians satisfaction (subjective parameters) in GPT-4o and human responses (blinded evaluations). Table D. Comparison of quality of citations (objective parameters) in GPTo1 and human responses (blinded evaluations). Table E. Comparison of physicians satisfaction (subjective parameters) in GPTo1 and human responses (blinded evaluations). Table F. Comparison of quality of citations (objective parameters) in OpenEvidence and human responses (blinded evaluations). Table G. Comparison of physicians satisfaction (subjective parameters) in OpenEvidence and human responses (blinded evaluations). Table H. Comparison of physicians satisfaction (subjective parameters) in all LLMs. Appendix A. The queries sent by the participating physicians. Appendix B. Resistance to change scale questionnaire. Section I: General information. Section II: Questionnaire Appendix C. Citation quality metrics used in the study. Appendix D. Satisfaction questionnaire used in the study.
(DOCX)

S1 Data Underlying data used for analysis. (XLS)
