# Supplementary material for: Real World Human-LLM Interactions – Prospective blinded versus unblinded expert physician assessments of LLM responses to complex medical dilemmas
Source: PLOS Digit Health. 2026 Mar 12;5(3):e0001278. doi: 10.1371/journal.pdig.0001278 (PMC12981447; doi:10.1371/journal.pdig.0001278)
Supplement: S1 Text — Fig B. Heat maps of Pearson correlation coefficients between physician satisfaction and citation quality metrics in unblinded evaluations. Table A. Comparison of blinded and unblinded physician satisfaction for each model. Table B. Comparison of quality of citations (objective parameters) in GPT-4o and human responses (blinded evaluations). Table C. Comparison of physicians satisfaction (subjective parameters) in GPT-4o and human responses (blinded evaluations). Table D. Comparison of quality of citations (objective parameters) in GPTo1 and human responses (blinded evaluations). Table E. Comparison of physicians satisfaction (subjective parameters) in GPTo1 and human responses (blinded evaluations). Table F. Comparison of quality of citations (objective parameters) in OpenEvidence and human responses (blinded evaluations). Table G. Comparison of physicians satisfaction (subjective parameters) in OpenEvidence and human responses (blinded evaluations). Table H. Comparison of physicians satisfaction (subjective parameters) in all LLMs. Appendix A. The queries sent by the participating physicians. Appendix B. Resistance to change scale questionnaire. Section I: General information. Section II: Questionnaire Appendix C. Citation quality metrics used in the study. Appendix D. Satisfaction questionnaire used in the study. (DOCX) [file pdig.0001278.s001.docx]

**Supplementary Material**

**Real World Human-LLM Interactions – Prospective Blinded versus Unblinded Expert Physician Assessments of LLM Responses to Complex Medical Dilemmas**

### Itamar Ben-Shitrit*^1,2^, Daphna Idan^1^, Mark Volevich^3^, Hadar Sharabi Goldenberg^4^, Dolev Vaknin^4^, Or Degany^4^, Nitzan Abelson^5^, Yair Binyamin^6^ Raouf Nassar^1,7^, Majd Nassar^5^, Aviya Kedmi^1^, Alexander Zlotnik^6^, Sharon Einav^8^

1 Ben-Gurion Faculty of Health Sciences, Beer-Sheva, Israel.

2 Clinical Research Center, Soroka University Medical Center, Faculty of Health Sciences, Ben-Gurion University of the Negev, Beer-Sheva 8410101, Israel

3 Technion - Israel Institute of Technology, Haifa, Israel

4 Faculty of Medicine, Tel-Aviv University, Israel

5 The Cheryl and Chaim Saban Children's Hospital, Soroka University Medical Center

6 Department of Anesthesia, Soroka University Medical Center and the Faculty of Health Sciences, Ben-Gurion University of the Negev, Beer-Sheva, Israel.

7 Pediatric Gastrointestinal Unit, Saban Children Hospital, Soroka University Medical Center, Beer-Sheva, Israel

8 Maccabi Healthcare Services and Hebrew University Faculty of Medicine, Israel.

**Corresponding Author:**

Itamar Ben-Shitrit,

E-mail: [itamab@post.bgu.ac.il](mailto:itamab@post.bgu.ac.il),

**Table of Contents**

[Fig A in S1_Text. Heat maps of Pearson correlation coefficients between physician satisfaction and citation quality metrics in blinded evaluations 3](#_Toc223088279)

[Fig B in S1_Text: Heat maps of Pearson correlation coefficients between physician satisfaction and citation quality metrics in unblinded evaluations. 4](#_Toc223088280)

[Table A in S1_Text. Comparison of blinded and unblinded physician satisfaction for each model. 7](#_Toc223088281)

[Table B in S1_Text. Comparison of quality of citations (objective parameters) in GPT-4o and human responses (blinded evaluations). 11](#_Toc223088282)

[Table C in S1_Text. Comparison of physicians satisfaction (subjective parameters) in GPT-4o and human responses (blinded evaluations). 13](#_Toc223088283)

[Table D in S1_Text. Comparison of quality of citations (objective parameters) in GPTo1 and human responses (blinded evaluations). 16](#_Toc223088284)

[Table E in S1_Text. Comparison of physicians satisfaction (subjective parameters) in GPTo1 and human responses (blinded evaluations). 18](#_Toc223088285)

[​​Table F in S1_Text. Comparison of quality of citations (objective parameters) in OpenEvidence and human responses (blinded evaluations). 21](#_Toc223088286)

[Table G in S1_Text. Comparison of physicians satisfaction (subjective parameters) in OpenEvidence and human responses (blinded evaluations). 23](#_Toc223088287)

[Table H in S1_Text. Comparison of physicians satisfaction (subjective parameters) in all LLMs 27](#_Toc223088288)

[Appendix 31](#_Toc223088289)

[Appendix A in S1_Text - The queries sent by the participating physicians. 31](#_Toc223088290)

[Appendix B in S1_Text - resistance to change scale questionnaire [1] 35](#_Toc223088291)

[Section I - General information 35](#_Toc223088292)

[Appendix C in S1_Text - Citation quality metrics used in the study. 36](#_Toc223088293)

[Appendix D in S1_Text - Satisfaction questionnaire used in the study. 37](#_Toc223088294)

## **Fig A in S1_Text. Heat maps of Pearson correlation coefficients between physician satisfaction and citation quality metrics in blinded evaluations**


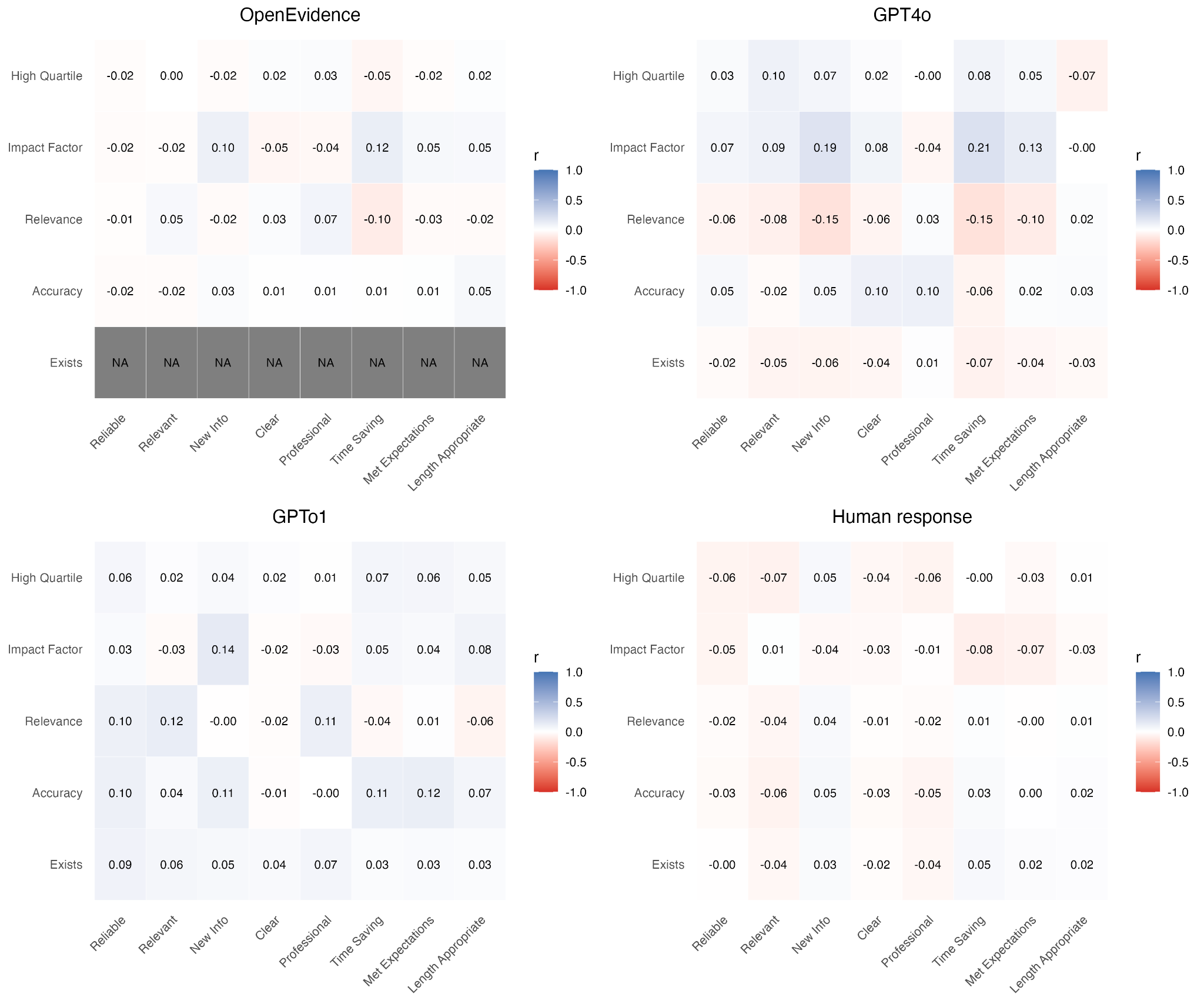


**Footnote:** Fig S1 in S1_Text. Heat-map of Pearson correlation coefficients ( r ) linking eight subjective satisfaction dimensions (columns) with five objective quality metrics (rows) for each source. Cell hues range from red (negative association) through white (no association) to blue (positive association); exact r values are overprinted. Grey cells marked “NA” indicate metrics that could not be computed for a given model.

## **Fig B in S1_Text: Heat maps of Pearson correlation coefficients between physician satisfaction and citation quality metrics in unblinded evaluations.**

**A.**


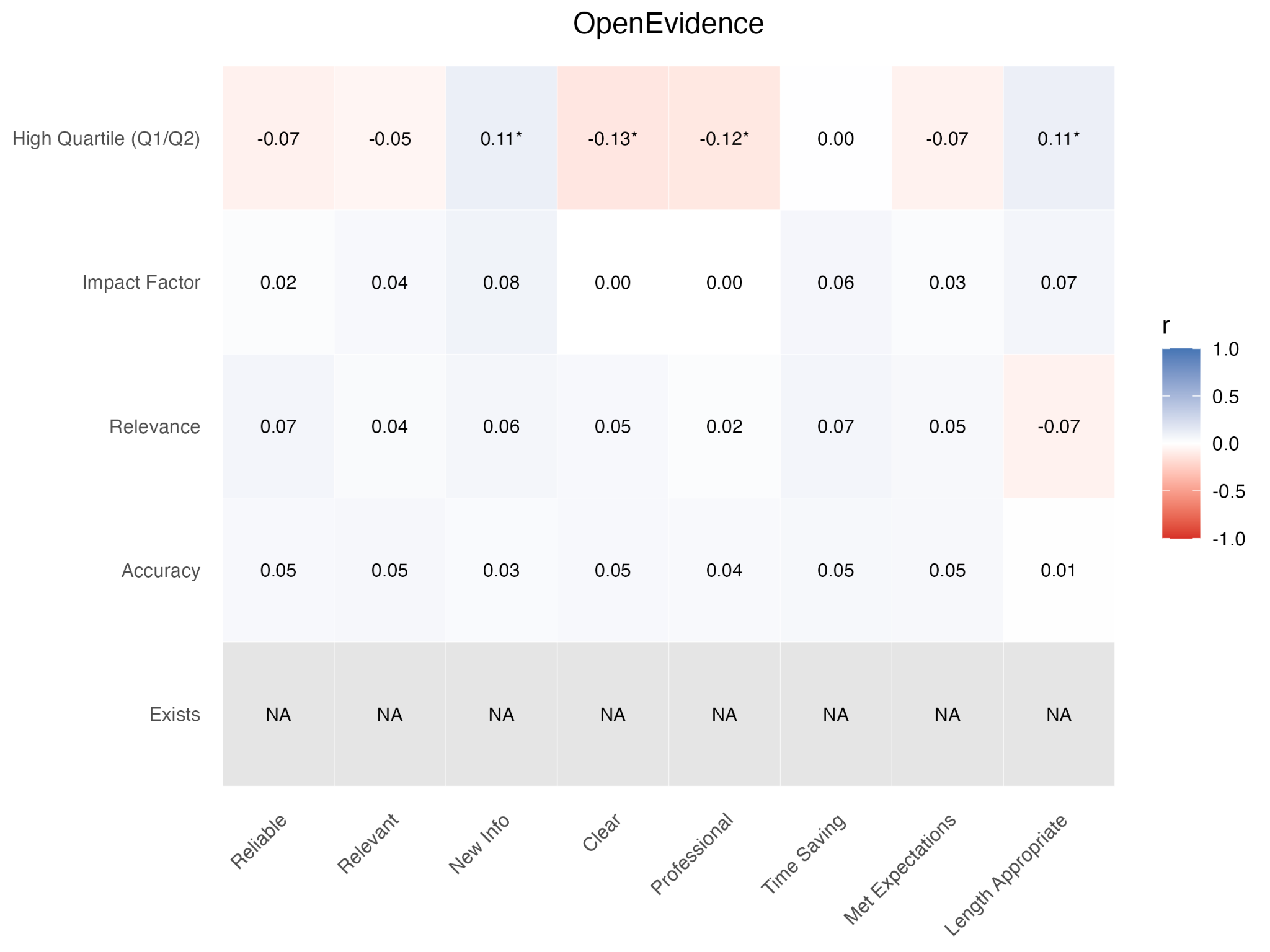


**B.**
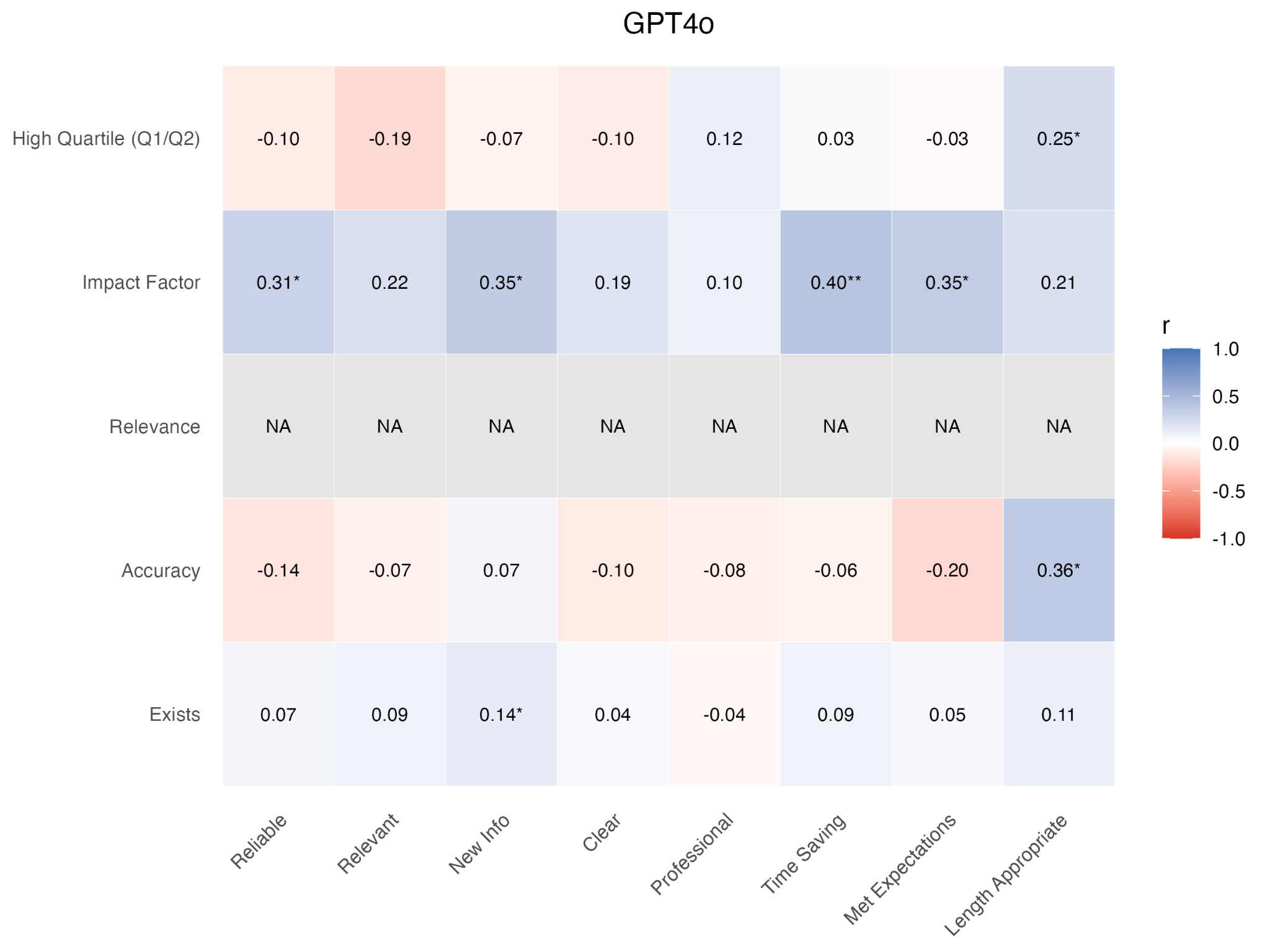


**C.**
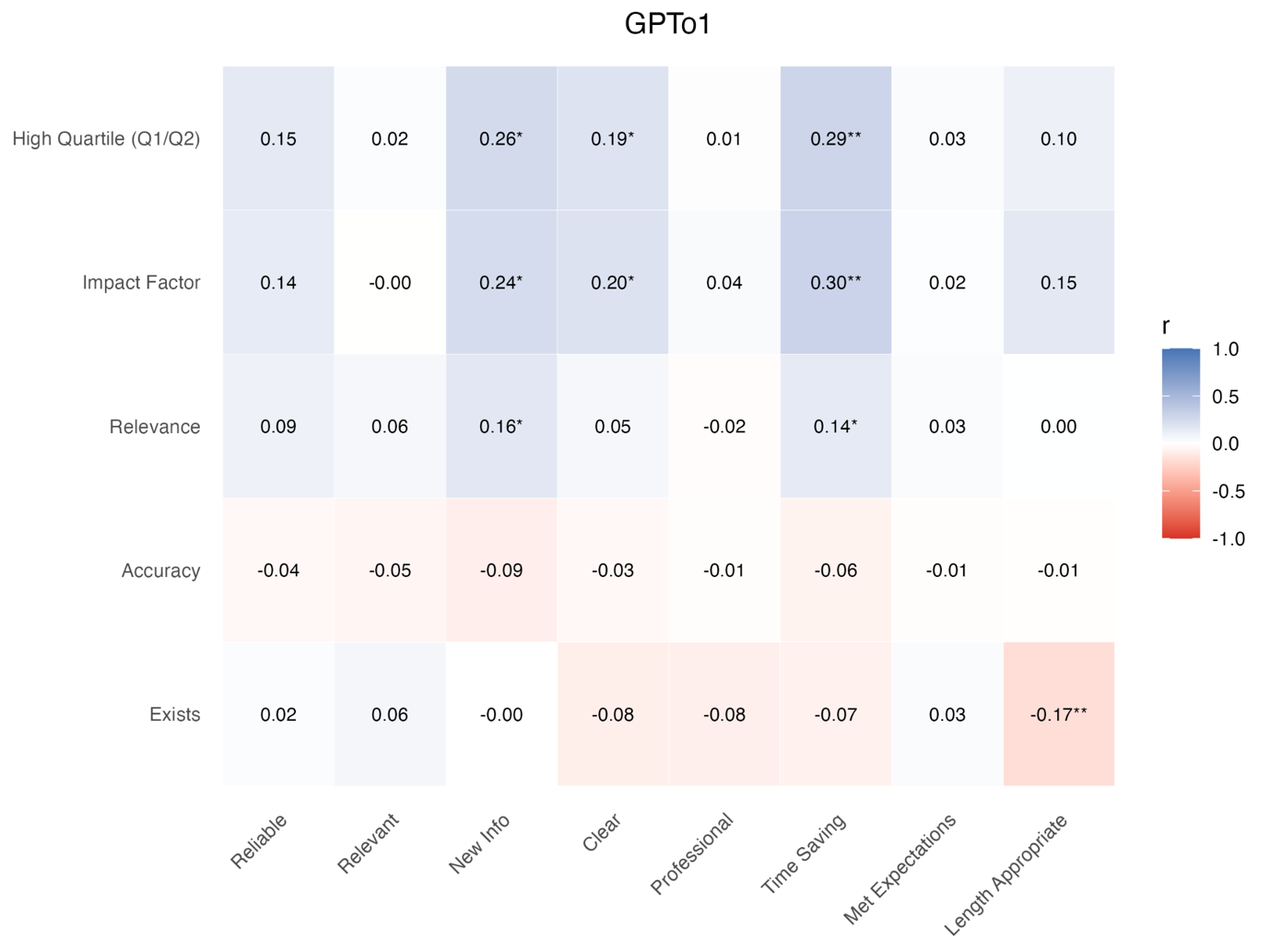


## **Table A in S1_Text. Comparison of blinded and unblinded physician satisfaction for each model.**

|  | **GPT4o** | | | **GPTo1** | | | **OpenEvidence** | | |
| --- | --- | --- | --- | --- | --- | --- | --- | --- | --- |
| **Characteristic** | **Blinded  N = 25^1^** | **Unblinded  N = 25^1^** | **p-value^2^** | **Blinded  N = 25^1^** | **Unblinded  N = 25^1^** | **p-value^2^** | **Blinded  N = 25^1^** | **Unblinded  N = 25^1^** | **p-value^2^** |
| The report is reliable |  |  | 0.001 |  |  | 0.4 |  |  | 0.8 |
| Strongly Agree | 2 (8.0%) | 7 (30%) |  | 4 (16%) | 2 (8.7%) |  | 5 (20%) | 7 (29%) |  |
| Agree | 20 (80%) | 7 (30%) |  | 20 (80%) | 16 (70%) |  | 16 (64%) | 13 (54%) |  |
| Neutral | 2 (8.0%) | 1 (4.3%) |  | 1 (4.0%) | 3 (13%) |  | 2 (8.0%) | 1 (4.2%) |  |
| Disagree | 1 (4.0%) | 4 (17%) |  | 0 (0%) | 1 (4.3%) |  | 2 (8.0%) | 3 (13%) |  |
| Strongly disagree | 0 (0%) | 4 (17%) |  | 0 (0%) | 1 (4.3%) |  |  |  |  |
| Information was relevant to medical inquiry |  |  | 0.4 |  |  | 0.8 |  |  | 0.6 |
| Strongly Agree | 8 (32%) | 9 (39%) |  | 7 (28%) | 9 (39%) |  | 8 (32%) | 9 (38%) |  |
| Agree | 11 (44%) | 7 (30%) |  | 15 (60%) | 13 (57%) |  | 12 (48%) | 11 (46%) |  |
| Neutral | 6 (24%) | 4 (17%) |  | 2 (8.0%) | 1 (4.3%) |  | 4 (16%) | 1 (4.2%) |  |
| Disagree | 0 (0%) | 1 (4.3%) |  | 1 (4.0%) | 0 (0%) |  | 1 (4.0%) | 2 (8.3%) |  |
| Strongly disagree | 0 (0%) | 2 (8.7%) |  |  |  |  | 0 (0%) | 1 (4.2%) |  |
| Report included new information |  |  | 0.5 |  |  | >0.9 |  |  | 0.9 |
| Strongly Agree | 1 (4.0%) | 1 (4.3%) |  | 2 (8.0%) | 2 (8.7%) |  | 2 (8.0%) | 3 (13%) |  |
| Agree | 6 (24%) | 8 (35%) |  | 8 (32%) | 6 (26%) |  | 7 (28%) | 4 (17%) |  |
| Neutral | 7 (28%) | 4 (17%) |  | 5 (20%) | 5 (22%) |  | 5 (20%) | 4 (17%) |  |
| Disagree | 9 (36%) | 5 (22%) |  | 8 (32%) | 7 (30%) |  | 9 (36%) | 10 (42%) |  |
| Strongly disagree | 2 (8.0%) | 5 (22%) |  | 2 (8.0%) | 3 (13%) |  | 2 (8.0%) | 3 (13%) |  |
| Information presented clearly |  |  | 0.2 |  |  | 0.5 |  |  | 0.2 |
| Strongly Agree | 3 (12%) | 3 (13%) |  | 6 (24%) | 7 (30%) |  | 4 (16%) | 9 (38%) |  |
| Agree | 20 (80%) | 13 (57%) |  | 17 (68%) | 11 (48%) |  | 19 (76%) | 12 (50%) |  |
| Neutral | 1 (4.0%) | 1 (4.3%) |  | 1 (4.0%) | 3 (13%) |  | 0 (0%) | 1 (4.2%) |  |
| Disagree | 1 (4.0%) | 2 (8.7%) |  | 1 (4.0%) | 2 (8.7%) |  | 2 (8.0%) | 2 (8.3%) |  |
| Strongly disagree | 0 (0%) | 4 (17%) |  |  |  |  |  |  |  |
| Report was professionally written |  |  | 0.2 |  |  | 0.3 |  |  | 0.4 |
| Strongly Agree | 5 (20%) | 2 (8.7%) |  | 10 (40%) | 6 (26%) |  | 8 (32%) | 10 (42%) |  |
| Agree | 18 (72%) | 13 (57%) |  | 10 (40%) | 14 (61%) |  | 14 (56%) | 8 (33%) |  |
| Neutral | 1 (4.0%) | 3 (13%) |  | 5 (20%) | 2 (8.7%) |  | 1 (4.0%) | 3 (13%) |  |
| Disagree | 1 (4.0%) | 2 (8.7%) |  | 0 (0%) | 1 (4.3%) |  | 2 (8.0%) | 3 (13%) |  |
| Strongly disagree | 0 (0%) | 3 (13%) |  |  |  |  |  |  |  |
| Report helped save time |  |  | 0.7 |  |  | >0.9 |  |  | 0.14 |
| Strongly Agree | 3 (12%) | 3 (13%) |  | 4 (16%) | 4 (17%) |  | 3 (12%) | 4 (17%) |  |
| Agree | 8 (32%) | 7 (30%) |  | 10 (40%) | 7 (30%) |  | 10 (40%) | 6 (25%) |  |
| Neutral | 6 (24%) | 3 (13%) |  | 6 (24%) | 6 (26%) |  | 5 (20%) | 7 (29%) |  |
| Disagree | 5 (20%) | 4 (17%) |  | 2 (8.0%) | 3 (13%) |  | 3 (12%) | 7 (29%) |  |
| Strongly disagree | 3 (12%) | 6 (26%) |  | 3 (12%) | 3 (13%) |  | 4 (16%) | 0 (0%) |  |
| Report met expectations |  |  | 0.068 |  |  | 0.5 |  |  | 0.5 |
| Strongly Agree | 1 (4.0%) | 6 (26%) |  | 3 (12%) | 3 (13%) |  | 4 (16%) | 8 (33%) |  |
| Agree | 10 (40%) | 5 (22%) |  | 11 (44%) | 8 (35%) |  | 7 (28%) | 5 (21%) |  |
| Neutral | 11 (44%) | 6 (26%) |  | 9 (36%) | 6 (26%) |  | 10 (40%) | 6 (25%) |  |
| Disagree | 3 (12%) | 4 (17%) |  | 2 (8.0%) | 6 (26%) |  | 4 (16%) | 4 (17%) |  |
| Strongly disagree | 0 (0%) | 2 (8.7%) |  |  |  |  | 0 (0%) | 1 (4.2%) |  |
| Report length was appropriate |  |  | 0.11 |  |  | 0.8 |  |  | 0.6 |
| Strongly Agree | 0 (0%) | 4 (17%) |  | 4 (16%) | 3 (13%) |  | 3 (12%) | 3 (13%) |  |
| Agree | 14 (56%) | 13 (57%) |  | 10 (40%) | 7 (30%) |  | 11 (44%) | 11 (46%) |  |
| Neutral | 3 (12%) | 1 (4.3%) |  | 4 (16%) | 7 (30%) |  | 1 (4.0%) | 4 (17%) |  |
| Disagree | 5 (20%) | 1 (4.3%) |  | 5 (20%) | 5 (22%) |  | 9 (36%) | 6 (25%) |  |
| Strongly disagree | 3 (12%) | 4 (17%) |  | 2 (8.0%) | 1 (4.3%) |  | 1 (4.0%) | 0 (0%) |  |
| **^1^n (%)**  **^2^**McNemar's test | | | | | | | | | |

## **Table B in S1_Text. Comparison of quality of citations (objective parameters) in GPT-4o and human responses (blinded evaluations).**

|  | **Human vs GPT4o** | |  |
| --- | --- | --- | --- |
| **Characteristic** | **GPT4o**  N = 361^1^ | **Manually generated human response**  N = 411^1^ | **p-value**^2^ |
| **Source verified (not-hallucinated)** | 352 (98%) | 409 (100%) | 0.019 |
| **Year accurate** | 222 (97%) | 369 (98%) | 0.5 |
| **Author accurate** | 222 (99%) | 375 (99%) | 0.7 |
| **Journal accurate** | 214 (97%) | 373 (99%) | 0.14 |
| **Faithful citation** | 98.6 (7.3) | 98.5 (9.8) | 0.83 |
| **Relevant** | 305 (88%) | 394 (97%) | <0.001 |
| **Publication type** |  |  | <0.001 |
| Book/Chapter | 4 (1.1%) | 4 (1.0%) |  |
| Case Reports/Series | 48 (13%) | 40 (9.7%) |  |
| Guidelines/Recommendations | 27 (7.5%) | 41 (10.0%) |  |
| Narrative Review | 31 (8.6%) | 97 (24%) |  |
| Observational Study | 55 (15%) | 98 (24%) |  |
| Other | 45 (12%) | 34 (8.3%) |  |
| Editorial | 6 (1.7%) | 4 (1.0%) |  |
| Randomized Controlled Trial | 10 (2.8%) | 37 (9.0%) |  |
| Systematic Review/Meta-analysis | 22 (6.1%) | 28 (6.8%) |  |
| Website | 113 (31%) | 28 (6.8%) |  |
| **Journal quartile** |  |  | 0.2 |
| 1 | 125 (63%) | 222 (63%) |  |
| 2 | 47 (24%) | 87 (25%) |  |
| 3 | 19 (9.6%) | 40 (11%) |  |
| 4 | 7 (3.5%) | 3 (0.9%) |  |
| **Impact factor** | 3.0 (1.8, 5.0) | 3.2 (1.9, 5.4) | 0.3 |
| ^1^n (%); Median (Q1, Q3); Mean (SD)  ^2^Pearson's Chi-squared test; Wilcoxon signed-rank test; T-test | | | |

​​

## **Table C in S1_Text. Comparison of physicians satisfaction (subjective parameters) in GPT-4o and human responses (blinded evaluations).**

|  | **Human vs GPT4o** | |  |
| --- | --- | --- | --- |
| **Characteristic** | **GPT4o  N = 25^1^** | **Manually generated human response  N = 25^1^** | **p-value^2^** |
| Report is reliable for complex medical questions |  |  | 0.080 |
| Strongly Agree | 2 (8.0%) | 7 (28%) |  |
| Agree | 20 (80%) | 18 (72%) |  |
| Neutral | 2 (8.0%) | 0 (0%) |  |
| Disagree | 1 (4.0%) | 0 (0%) |  |
| Strongly Disagree | – | – |  |
| Information was relevant to medical inquiry |  |  | 0.2 |
| Strongly Agree | 8 (32%) | 13 (52%) |  |
| Agree | 11 (44%) | 9 (36%) |  |
| Neutral | 6 (24%) | 2 (8.0%) |  |
| Disagree | 0 (0%) | 1 (4.0%) |  |
| Strongly Disagree | – | – |  |
| Report included new information |  |  | 0.3 |
| Strongly Agree | 1 (4.0%) | 5 (20%) |  |
| Agree | 6 (24%) | 8 (32%) |  |
| Neutral | 7 (28%) | 6 (24%) |  |
| Disagree | 9 (36%) | 6 (24%) |  |
| Strongly disagree | 2 (8.0%) | 0 (0%) |  |
| Information presented clearly |  |  | 0.051 |
| Strongly Agree | 3 (12%) | 10 (40%) |  |
| Agree | 20 (80%) | 15 (60%) |  |
| Neutral | 1 (4.0%) | 0 (0%) |  |
| Disagree | 1 (4.0%) | 0 (0%) |  |
| Strongly Disagree | – | – |  |
| Report was professionally written |  |  | 0.035 |
| Strongly Agree | 5 (20%) | 13 (52%) |  |
| Agree | 18 (72%) | 10 (40%) |  |
| Neutral | 1 (4.0%) | 2 (8.0%) |  |
| Disagree | 1 (4.0%) | 0 (0%) |  |
| Strongly Disagree | – | – |  |
| Report helped save time |  |  | 0.4 |
| Strongly Agree | 3 (12%) | 5 (20%) |  |
| Agree | 8 (32%) | 11 (44%) |  |
| Neutral | 6 (24%) | 4 (16%) |  |
| Disagree | 5 (20%) | 1 (4.0%) |  |
| Strongly disagree | 3 (12%) | 4 (16%) |  |
| Report met expectations |  |  | 0.11 |
| Strongly Agree | 1 (4.0%) | 7 (28%) |  |
| Agree | 10 (40%) | 9 (36%) |  |
| Neutral | 11 (44%) | 8 (32%) |  |
| Disagree | 3 (12%) | 1 (4.0%) |  |
| Strongly Disagree | – | – |  |
| Report length was appropriate |  |  | 0.071 |
| Strongly Agree | 0 (0%) | 5 (20%) |  |
| Agree | 14 (56%) | 10 (40%) |  |
| Neutral | 3 (12%) | 1 (4.0%) |  |
| Disagree | 5 (20%) | 8 (32%) |  |
| Strongly disagree | 3 (12%) | 1 (4.0%) |  |
| ^1^n (%)  ^2^McNemar's test | | | |

## **Table D in S1_Text. Comparison of quality of citations (objective parameters) in GPTo1 and human responses (blinded evaluations).**

|  | Human vs GPTo1 | |  |
| --- | --- | --- | --- |
| **Characteristic** | **GPTo1  N = 441^1^** | **Manually generated human response**  **N = 411^1^** | **p-value^2^** |
| **Source verified (not-hallucinated)** | 154 (35%) | 409 (100%) | <0.001 |
| **Year accurate** | 100 (72%) | 369 (98%) | <0.001 |
| **Author accurate** | 112 (82%) | 375 (99%) | <0.001 |
| **Journal accurate** | 113 (78%) | 373 (99%) | <0.001 |
| **Faithful citation** | 76.7 (35.8) | 98.5 (9.8) | <0.001 |
| **Relevant** | 107 (77%) | 394 (97%) | <0.001 |
| **Publication type** |  |  | <0.001 |
| **Book/Chapter** | 12 (2.7%) | 4 (1.0%) |  |
| **Case Reports/Series** | 2 (0.5%) | 40 (9.7%) |  |
| **Guidelines/Recommendations** | 46 (10%) | 41 (10.0%) |  |
| **Narrative Review** | 39 (8.8%) | 97 (24%) |  |
| **Observational Study** | 24 (5.4%) | 98 (24%) |  |
| **Other** | 299 (68%) | 34 (8.3%) |  |
| **Editorial** | 0 (0%) | 4 (1.0%) |  |
| **Randomized Controlled Trial** | 1 (0.2%) | 37 (9.0%) |  |
| **Systematic Review/Meta-analysis** | 7 (1.6%) | 28 (6.8%) |  |
| **Website** | 11 (2.5%) | 28 (6.8%) |  |
| **Journal quartile** |  |  | <0.001 |
| **1** | 97 (84%) | 222 (63%) |  |
| **2** | 11 (9.5%) | 87 (25%) |  |
| **3** | 5 (4.3%) | 40 (11%) |  |
| **4** | 3 (2.6%) | 3 (0.9%) |  |
| **Impact factor** | 4.4 (2.4, 7.5) | 3.2 (1.9, 5.4) | <0.001 |
| ^1^n (%); Median (Q1, Q3); Mean (SD)  ^2^Pearson's Chi-squared test; Wilcoxon signed-rank test; T-test | | | |

​​

## **Table E in S1_Text. Comparison of physicians satisfaction (subjective parameters) in GPTo1 and human responses (blinded evaluations).**

|  | **Human vs GPTo1** | |  |
| --- | --- | --- | --- |
| **Characteristic** | **GPTo1  N = 25^1^** | **Manually generated human response  N = 25^1^** | **p-value^2^** |
| Report is reliable for complex medical questions |  |  | 0.5 |
| Strongly Agree | 4 (16%) | 7 (28%) |  |
| Agree | 20 (80%) | 18 (72%) |  |
| Neutral | 1 (4.0%) | 0 (0%) |  |
| Disagree | – | – |  |
| Strongly Disagree | – | – |  |
| Information was relevant to medical inquiry |  |  | 0.3 |
| Strongly Agree | 7 (28%) | 13 (52%) |  |
| Agree | 15 (60%) | 9 (36%) |  |
| Neutral | 2 (8.0%) | 2 (8.0%) |  |
| Disagree | 1 (4.0%) | 1 (4.0%) |  |
| Strongly Disagree | – | – |  |
| Report included new information |  |  | 0.5 |
| Strongly Agree | 2 (8.0%) | 5 (20%) |  |
| Agree | 8 (32%) | 8 (32%) |  |
| Neutral | 5 (20%) | 6 (24%) |  |
| Disagree | 8 (32%) | 6 (24%) |  |
| Strongly disagree | 2 (8.0%) | 0 (0%) |  |
| Information presented clearly |  |  | 0.4 |
| Strongly Agree | 6 (24%) | 10 (40%) |  |
| Agree | 17 (68%) | 15 (60%) |  |
| Neutral | 1 (4.0%) | 0 (0%) |  |
| Disagree | 1 (4.0%) | 0 (0%) |  |
| Strongly Disagree | – | – |  |
| Report was professionally written |  |  | 0.5 |
| Strongly Agree | 10 (40%) | 13 (52%) |  |
| Agree | 10 (40%) | 10 (40%) |  |
| Neutral | 5 (20%) | 2 (8.0%) |  |
| Disagree | – | – |  |
| Strongly Disagree | – | – |  |
| Report helped save time |  |  | >0.9 |
| Strongly Agree | 4 (16%) | 5 (20%) |  |
| Agree | 10 (40%) | 11 (44%) |  |
| Neutral | 6 (24%) | 4 (16%) |  |
| Disagree | 2 (8.0%) | 1 (4.0%) |  |
| Strongly disagree | 3 (12%) | 4 (16%) |  |
| Report met expectations |  |  | 0.6 |
| Strongly Agree | 3 (12%) | 7 (28%) |  |
| Agree | 11 (44%) | 9 (36%) |  |
| Neutral | 9 (36%) | 8 (32%) |  |
| Disagree | 2 (8.0%) | 1 (4.0%) |  |
| Strongly Disagree | – | – |  |
| Report length was appropriate |  |  | 0.6 |
| Strongly Agree | 4 (16%) | 5 (20%) |  |
| Agree | 10 (40%) | 10 (40%) |  |
| Neutral | 4 (16%) | 1 (4.0%) |  |
| Disagree | 5 (20%) | 8 (32%) |  |
| Strongly disagree | 2 (8.0%) | 1 (4.0%) |  |
| ^1^n (%)  ^2^McNemar's test | | | |

## **​​Table F in S1_Text. Comparison of quality of citations (objective parameters) in OpenEvidence and human responses (blinded evaluations).**

|  | **Human vs OpenEvidence** | |  |
| --- | --- | --- | --- |
| **Characteristic** | **Manually generated human response**  N = 411^1^ | **OpenEvidence**  N = 542^1^ | **p-value**^2^ |
| **Source verified (not-hallucinated)** | 409 (100%) | 540 (100%) | 0.2 |
| **Relevant** | 394 (97%) | 498 (92%) | 0.002 |
| **Publication type** |  |  | <0.001 |
| Book/Chapter | 4 (1.0%) | 5 (0.9%) |  |
| Case Reports/Series | 40 (9.7%) | 49 (9.0%) |  |
| Guidelines/Recommendations | 41 (10.0%) | 78 (14%) |  |
| Narrative Review | 97 (24%) | 157 (29%) |  |
| Observational Study | 98 (24%) | 153 (28%) |  |
| Other | 34 (8.3%) | 20 (3.7%) |  |
| Editorial | 4 (1.0%) | 0 (0%) |  |
| Randomized Controlled Trial | 37 (9.0%) | 24 (4.4%) |  |
| Systematic Review/Meta-analysis | 28 (6.8%) | 44 (8.1%) |  |
| Website | 28 (6.8%) | 12 (2.2%) |  |
| **Journal quartile** |  |  | <0.001 |
| 1 | 222 (63%) | 400 (78%) |  |
| 2 | 87 (25%) | 81 (16%) |  |
| 3 | 40 (11%) | 24 (4.7%) |  |
| 4 | 3 (0.9%) | 8 (1.6%) |  |
| **Impact factor** | 3.2 (1.9, 5.4) | 3.8 (2.5, 5.9) | 0.002 |
| ^1^n (%); Median (Q1, Q3); Mean (SD)  ^2^Pearson's Chi-squared test; Wilcoxon signed-rank test; T-test | | | |

## **Table G in S1_Text. Comparison of physicians satisfaction (subjective parameters) in OpenEvidence and human responses (blinded evaluations).**

|  | **Human vs OpenEvidence** | |  |
| --- | --- | --- | --- |
| **Characteristic** | **Manually generated human response**  **N = 25^1^** | **OpenEvidence  N = 25^1^** | **p-value^2^** |
| Report is reliable for complex medical questions |  |  | 0.3 |
| Strongly Agree | 7 (28%) | 5 (20%) |  |
| Agree | 18 (72%) | 16 (64%) |  |
| Neutral | 0 (0%) | 2 (8.0%) |  |
| Disagree | 0 (0%) | 2 (8.0%) |  |
| Strongly Disagree | – | – |  |
| Information was relevant to medical inquiry |  |  | 0.5 |
| Strongly Agree | 13 (52%) | 8 (32%) |  |
| Agree | 9 (36%) | 12 (48%) |  |
| Neutral | 2 (8.0%) | 4 (16%) |  |
| Disagree | 1 (4.0%) | 1 (4.0%) |  |
| Strongly Disagree | – | – |  |
| Report included new information |  |  | 0.5 |
| Strongly Agree | 5 (20%) | 2 (8.0%) |  |
| Agree | 8 (32%) | 7 (28%) |  |
| Neutral | 6 (24%) | 5 (20%) |  |
| Disagree | 6 (24%) | 9 (36%) |  |
| Strongly disagree | 0 (0%) | 2 (8.0%) |  |
| Information presented clearly |  |  | 0.070 |
| Strongly Agree | 10 (40%) | 4 (16%) |  |
| Agree | 15 (60%) | 19 (76%) |  |
| Disagree | 0 (0%) | 2 (8.0%) |  |
|  |  |  |  |
| Disagree | – | – |  |
| Strongly Disagree | – | – |  |
| Report was professionally written |  |  | 0.3 |
| Agree | 10 (40%) | 14 (56%) |  |
| Disagree | 0 (0%) | 2 (8.0%) |  |
| Neutral | 2 (8.0%) | 1 (4.0%) |  |
| Strongly Agree | 13 (52%) | 8 (32%) |  |
| Report helped save time |  |  | 0.9 |
| Agree | 11 (44%) | 10 (40%) |  |
| Disagree | 1 (4.0%) | 3 (12%) |  |
| Neutral | 4 (16%) | 5 (20%) |  |
| Strongly Agree | 5 (20%) | 3 (12%) |  |
| Strongly disagree | 4 (16%) | 4 (16%) |  |
| Report met expectations |  |  | 0.4 |
| Agree | 9 (36%) | 7 (28%) |  |
| Disagree | 1 (4.0%) | 4 (16%) |  |
| Neutral | 8 (32%) | 10 (40%) |  |
| Strongly Agree | 7 (28%) | 4 (16%) |  |
| Report length was appropriate |  |  | >0.9 |
| Agree | 10 (40%) | 11 (44%) |  |
| Disagree | 8 (32%) | 9 (36%) |  |
| Neutral | 1 (4.0%) | 1 (4.0%) |  |
| Strongly Agree | 5 (20%) | 3 (12%) |  |
| Strongly disagree | 1 (4.0%) | 1 (4.0%) |  |
| ^1^n (%)  ^2^McNemar's test | | | |

| **Table H in S1_Text. Comparison of physicians satisfaction (subjective parameters) in all LLMs** | | | | |
| --- | --- | --- | --- | --- |
|  | **Unblinded evaluation** | | | |
| Characteristic | GPT4o  N = 25^1^ | GPTo1  N = 25^1^ | OpenEvidence  N = 25^1^ | p-value^2^ |
| Report is reliable for complex medical questions |  |  |  | 0.042 |
| Strongly Agree | 7 (30%) | 2 (8.7%) | 7 (29%) |  |
| Agree | 7 (30%) | 16 (70%) | 13 (54%) |  |
| Disagree | 4 (17%) | 1 (4.3%) | 3 (13%) |  |
| Neutral | 1 (4.3%) | 3 (13%) | 1 (4.2%) |  |
| Strongly disagree | 4 (17%) | 1 (4.3%) | 0 (0%) |  |
| Information was relevant to medical inquiry |  |  |  | 0.4 |
| Strongly Agree | 9 (39%) | 9 (39%) | 9 (38%) |  |
| Agree | 7 (30%) | 13 (57%) | 11 (46%) |  |
| Neutral | 4 (17%) | 1 (4.3%) | 1 (4.2%) |  |
| Disagree | 1 (4.3%) | 0 (0%) | 2 (8.3%) |  |
| Strongly disagree | 2 (8.7%) | 0 (0%) | 1 (4.2%) |  |
| Report included new information |  |  |  | 0.8 |
| Strongly Agree | 1 (4.3%) | 2 (8.7%) | 3 (13%) |  |
| Agree | 8 (35%) | 6 (26%) | 4 (17%) |  |
| Neutral | 4 (17%) | 5 (22%) | 4 (17%) |  |
| Disagree | 5 (22%) | 7 (30%) | 10 (42%) |  |
| Strongly disagree | 5 (22%) | 3 (13%) | 3 (13%) |  |
| Information presented clearly |  |  |  | 0.2 |
| Strongly Agree | 3 (13%) | 7 (30%) | 9 (38%) |  |
| Agree | 13 (57%) | 11 (48%) | 12 (50%) |  |
| Neutral | 1 (4.3%) | 3 (13%) | 1 (4.2%) |  |
| Disagree | 2 (8.7%) | 2 (8.7%) | 2 (8.3%) |  |
| Strongly disagree | 4 (17%) | 0 (0%) | 0 (0%) |  |
| Report was professionally written |  |  |  | 0.081 |
| Strongly Agree | 2 (8.7%) | 6 (26%) | 10 (42%) |  |
| Agree | 13 (57%) | 14 (61%) | 8 (33%) |  |
| Neutral | 3 (13%) | 2 (8.7%) | 3 (13%) |  |
| Disagree | 2 (8.7%) | 1 (4.3%) | 3 (13%) |  |
| Strongly disagree | 3 (13%) | 0 (0%) | 0 (0%) |  |
| Report helped save time |  |  |  | 0.3 |
| Strongly Agree | 3 (13%) | 4 (17%) | 4 (17%) |  |
| Agree | 7 (30%) | 7 (30%) | 6 (25%) |  |
| Neutral | 3 (13%) | 6 (26%) | 7 (29%) |  |
| Disagree | 4 (17%) | 3 (13%) | 7 (29%) |  |
| Strongly disagree | 6 (26%) | 3 (13%) | 0 (0%) |  |
| Report met expectations |  |  |  | 0.7 |
| Strongly Agree | 6 (26%) | 3 (13%) | 8 (33%) |  |
| Agree | 5 (22%) | 8 (35%) | 5 (21%) |  |
| Neutral | 6 (26%) | 6 (26%) | 6 (25%) |  |
| Disagree | 4 (17%) | 6 (26%) | 4 (17%) |  |
| Strongly disagree | 2 (8.7%) | 0 (0%) | 1 (4.2%) |  |
| Report length was appropriate |  |  |  | 0.050 |
| Strongly Agree | 4 (17%) | 3 (13%) | 3 (13%) |  |
| Agree | 13 (57%) | 7 (30%) | 11 (46%) |  |
| Neutral | 1 (4.3%) | 7 (30%) | 4 (17%) |  |
| Disagree | 1 (4.3%) | 5 (22%) | 6 (25%) |  |
| Strongly disagree | 4 (17%) | 1 (4.3%) | 0 (0%) |  |
| ^1^n (%)  ^2^Friedman test | | | | |

# Appendix

## **Appendix A in S1_Text - The queries sent by the participating physicians.**

| **Physician specialty \ query serial number** | **1** | **2** | **3** | **4** | **5** |
| --- | --- | --- | --- | --- | --- |
| **Pediatrics** | A 3-month-old male patient with chronic granulomatous disease without complications at the moment.  What are the considerations for and against stem cell transplant in a 3-month-old male patient with chronic granulomatous disease without complications at the moment? Could I have some literature cases and reviews? | A 12-year-old boy who presents with frequent sinusitis and 2 different events of lobar pneumonia, who underwent routine vaccinations.  When looking into assessing the immune function of the patient, which serology tests have been verified as most useful to detect an abnormal or insufficient immune response to vaccines and why? | A 15-year-old male patient with suspected eosinophilic esophagitis due to weight loss and progressive dysphagia. What are treatment options after a positive gastroscopy and biopsy, and how to assess for treatment failure in these cases? | A 12-year-old female with poorly controlled asthma. Compare the efficacy of Flutiform, Smbicort, and Foster inhalers on asthma control, and the different dosage possibilities for her. | A comparison of the long-term (over 10 years daily use) use of oral antihistamines versus nasal antihistamines versus nasal glucocorticoids for allergic rhinitis in patients starting treatment before age 10. |
| **Pediatric gastroenterology** | A 12-year-old girl swallowed a "Hijab pin" 2 weeks ago. Her X-ray and abdominal CT showed the pin near the appendix. The girl is asymptomatic. What is the risk of having acute appendicitis if you do not remove this pin? | A one-day-old baby child was born without any complications. For unknown reasons, the resident took a blood test which revealed elevated GGT and slightly elevated ALT and AST. The US showed a normal gallbladder. What is the risk for this patient to have biliary atresia, and do you recommend the patient undergo an intraoperative cholangiography at this age? | A 6-year-old child suffering from Autosomal recessive polycystic kidney disease. Due to progressive thrombocytopenia, he underwent an upper gastroendoscopy, which revealed two esophageal varices grade 2. What is the benefit of starting a prophylactic beta blocker for this child? | A 14-year-old girl has been suffering from elevated liver enzymes for more than half a year. Her BMI is 97th percentile. The autoimmune, infectious, and metabolic tests were normal. Abdominal US revealed a heterogenic enlarged liver. Assuming that the patient suffers from metabolic dysfunction-associated steatotic liver disease (MASLD), what is the chance (in %) of normalizing her liver enzymes within six months if she changes her lifestyle? | six-month-old girl was admitted to the PER with a fever of up to 39.5°C for the last 12 hours without any other symptoms. Her blood tests revealed leukocytosis (17,000) with neutrophilia (10,000), and the CRP was slightly elevated (6; normal up to 0.5). Her urine stick was normal. Assuming she is vaccinated, what is the chance that she has occult bacteremia? Should she be given empiric antibiotic treatment? |
| **Obstetric anesthesia** | A 30-year-old patient, in her fourth pregnancy, was brought in for an emergency cesarean section due to placental abruption and fetal distress. Spinal anesthesia was administered using a spinal needle (22G/27G). Upon needle withdrawal, the anesthesiologist noticed that the distal tip had broken off and remained inside the patient's body. It was decided to proceed with the urgent surgery. Postoperatively, the patient was sent for a CT scan, which revealed a 1 cm fragment of the needle lodged partially in the dura, with part in the spinal canal and part in the epidural space. The patient is asymptomatic, and the question arises: should surgical removal of the needle fragment be pursued, or is it preferable to leave it in place? I would appreciate your insights on the considerations for each approach and your recommendation regarding the timing of potential surgery. | A 24-year-old primigravid patient at 35 weeks of gestation presented with shortness of breath during pregnancy. She underwent a CT angiography to rule out pulmonary embolism, which was excluded. However, a large echinococcal cyst was identified in the liver, and additional findings in the lung suggested the presence of pulmonary arteriovenous malformation (AVM). On physical examination, she was not dyspneic at rest, but her room air oxygen saturation was 91%, without accessory muscle use. Nail clubbing was noted on inspection of her hands. A brain CT scan was normal, with no evidence of echinococcosis. Echocardiography showed preserved cardiac function, but a bubble study confirmed the diagnosis of pulmonary AVM. What is the recommended obstetric and anesthetic approach for a patient with a liver echinococcal cyst?  What is the recommended obstetric and anesthetic approach for a patient with a pulmonary arteriovenous malformation? | A 30-year-old female patient with Neurofibromatosis Type 1 presents for an elective cesarean section due to a history of two previous cesarean deliveries. She is currently not on any chronic medications. Physical examination reveals numerous cutaneous neurofibromas (hundreds). Notably, she underwent an adrenalectomy four years ago due to pheochromocytoma without any complications. The patient had a brain and spinal cord MRI six years ago.  The primary question concerns the feasibility of performing spinal anesthesia on this patient. Is updated imaging necessary due to the potential for central nervous system tumors, and would a six-year-old imaging study be sufficient, or is more recent imaging required? If the latter, how many years prior should the imaging be performed to be considered adequate? | A 35-year-old G2P1 patient with multiple sclerosis (MS) presents in early labor. She has a relapsing-remitting form of MS, with her last flare-up occurring three years ago, followed by a full recovery. The patient expresses a strong preference for epidural analgesia during labor. Neurologically, she is stable, with no current symptoms, and her MS has been well-managed with disease-modifying therapy, which was discontinued during pregnancy.  Is epidural analgesia safe in this patient, or could it trigger an MS flare-up? What factors should be considered when determining the appropriate analgesic or anesthetic approach for labor in a patient with multiple sclerosis, and is there a need for special monitoring or precautions during the procedure? | A 38-year-old female, weighing 165 kg with a height of 1.59 meters, presents for her first delivery following fertility treatments. She also has scoliosis, for which she has not undergone corrective surgery. The patient is a candidate for a vaginal delivery. During an epidural attempt, multiple attempts were made, resulting in an accidental dural puncture. Should another epidural attempt be made, or would it be preferable to place a spinal catheter and manage the labor with continuous spinal analgesia?  Additionally, after delivery, could treatment with cosyntropin reduce the risk of developing post-dural puncture headache (PDPH) or mitigate its severity? |
| **Pediatric endocrinology** | A 17.7-year-old female presents with abdominal pain for 3 weeks. The pain is located in the lower abdomen and is intermittent. She has also experienced weight loss, hyperpigmentation in the face and fingers, and fatigue. Laboratory investigations reveal primary hypothyroidism and primary adrenal insufficiency. The patient has a family history of Addison's disease, with no known medication or substance use. How should she be managed? | A 3-year-old male with a history of bilateral cryptorchidism, who underwent orchiopexy at age 2, presents for an initial endocrine evaluation. Physical examination reveals a small testicular size of 1-2 ml bilaterally. What are the potential implications for future fertility and hormonal development, and how should long-term follow-up be structured for this patient? | A 4-year-old male patient with a confirmed diagnosis of Aromatase Excess Syndrome is currently under the care of an endocrinologist. Given his current height percentile of 90, what are the recommended therapeutic interventions to optimize his growth trajectory and achieve a normal adult height? | A 7-month-old unvaccinated infant presents to the emergency department with a 3-day history of high fever. On examination, the infant appears lethargic with decreased responsiveness and a bulging fontanelle. A lumbar puncture is performed, revealing cloudy cerebrospinal fluid. Laboratory tests demonstrate an elevated inflammatory marker, with elevated C-reactive protein (CRP). What is the immediate treatment that should be administered to this child? | A 10-year-old patient with a heterozygous ABCC8 gene mutation presents with hypoglycemic events, despite frequent meals including overnight PEG feeding. Sensor data reveals low sugar values 10% of the time. What are the recommended treatment options for this patient? |
| **Internal medicine** | A 34-year-old patient with DLBCL who presented with a picture of CNS infection with zero cells in the CSF, a Biofire test of the fluid is positive for herpes simplex 1. What is the incidence of herpes simplex encephalitis with a normal cell count? | A healthy 48-year-old male presenting with intussusception was diagnosed with celiac disease. What is the prevalence of celiac disease as a cause of intussusception in adults? | A 70-year-old male with a diagnosis of GCA (Giant Cell Arteritis). Shortly after treatment with Actemra (Tocilizumab) was initiated - the patient was diagnosed with PE.  Does the use of Tocilizumab (Actemra) increase the risk of thrombosis? | 64 years old with a background of chronic metabolic syndrome and ischemic heart disease who received Venofer as part of hospitalization and 3 days later developed acute hepatitis.  Can intravenous iron formulation cause acute liver injury in a healthy individual? | A 26-year-old healthy woman, who experienced pregnancy-related hypertension and HELLP syndrome 10 months ago, has had two positive triple APLA tests since then. She is presenting with chest pain and shortness of breath, clinical suspicion of PE was raised, which was not demonstrated by a chest CT scan.  Is thromboprophylaxis recommended in carriers of antiphospholipid antibodies without prior thrombosis |

## **Appendix B in S1_Text - resistance to change scale questionnaire [1]**

### Section I - General information

- Age
- Gender
- Family status
- Religion
- Country of birth

Section II - Questionnaire
Respond to the question using a 1-6 scale, where:
 1 = Strongly Disagree
 2 = Disagree
 3 = Somewhat Disagree
 4 = Somewhat Agree
 5 = Agree
 6 = Strongly Agree

- I generally consider changes to be a negative thing
- I'll take a routine day over a dau full of unexpected events any time
- I like to do the same old things rather than try new and different ones.
- Whenever my life forms a stable routine, I look for ways to change it.
- I'd rather be bored than surprised
- If I were to be informed that there is going to be a significant change regarding the way things are done at school, I would probably feel stressed.
- When I am informed of a change of plans, I tense up a bit.
- When things don't go according to plans, it stresses me out.
- If one of my professors changed the grading criteria, it would probably make me feel uncomfortable even if I thought I'd do just as well without having to do any extra work.
- Changing plans seems like a real hassle to me.
- Often, I feel a bit uncomfortable even about changes that may potentially improve my life.
- When someone pressures me to change something, I tend to resist it even if I think the change may ultimately benefit me.
- I sometimes find myself avoiding changes that I know will be good for me.
- I often change my mind.
- I don't change my mind easily.
- Once I've come to a conclusion, I'm not likely to change my mind.
- My views are very consistent over time.

## **Appendix C in S1_Text - Citation quality metrics used in the study.**

- Citation validation
  - Does the citation exist? [2-3]
    - cited journal, title, PubmedID number, authors, publication year, volume, issue, and pages.
  - Does the text generated comply with the original text? [4]
  - Does the cited source provide evidence that is relevant to the clinical dilemma?

- Citation type
  - Book/Chapter
  - Case Reports/Series
  - Guidelines/Recommendations
  - Narrative Review
  - Observational Study
  - Randomized Controlled Trial
  - Systematic Review/Meta-analysis
  - Website
  - Editorial
  - Other
- Citation quantitative metrics
  - Impact factor
  - Quartile
- Number of citations

## **Appendix D in S1_Text - Satisfaction questionnaire used in the study.**

Please rate the following statements based on your experience with the report, using a scale from 'Strongly Disagree' to 'Strongly Agree’.

**1 -** Strongly disagree**, 2 -** Disagree**, 3 -** Neutral**, 4 -** Agree**, 5 -** Strongly agree

- The report generated by a technological tool\human researcher is reliable for answering complex medical questions.
- The information provided in the report was relevant to my medical inquiry.
- The report included new information that was previously unknown to me or my group.
- The information in the report was presented in a clear and understandable way.
- The report was professionally written.
- The information provided in the report helped save time for me or my group.
- The report met my expectations.
- The length of the report was appropriate.
- The report could have been improved. If you agree, please share your suggestions in the "Other" section.

**References**

1. Oreg, S. Resistance to change: Developing an individual differences measure. *Journal of Applied Psychology* **88**, 680–693 (2003).
2. Chen, A. & Chen, D. O. Accuracy of Chatbots in Citing Journal Articles. *JAMA Netw Open* **6**, e2327647 (2023).
3. Walters, W. H. & Wilder, E. I. Fabrication and errors in the bibliographic citations generated by ChatGPT. *Sci Rep* **13**, 14045 (2023).
4. Cascella, M., Montomoli, J., Bellini, V. & Bignami, E. Evaluating the Feasibility of ChatGPT in Healthcare: An Analysis of Multiple Clinical and Research Scenarios. *J Med Syst* **47**, 33 (2023).
